# Sternum Metastases: From Case-Identifying Strategy to Multidisciplinary Management

**DOI:** 10.3390/diagnostics13162698

**Published:** 2023-08-17

**Authors:** Mara Carsote, Dana Terzea, Florina Vasilescu, Anca-Pati Cucu, Adrian Ciuche, Claudiu Nistor

**Affiliations:** 1 Department of Endocrinology, Carol Davila University of Medicine and Pharmacy, 020021 Bucharest, Romania; carsote_m@hotmail.com; 2 Department of Endocrinology, C.I. Parhon National Institute of Endocrinology, 020021 Bucharest, Romania; 3 Department of Pathology, C.I. Parhon National Institute of Endocrinology, 020021 Bucharest, Romania; danaterzea@gmail.com; 4 Department of Pathology, Dr. Carol Davila Central Military Emergency University Hospital, 020021 Bucharest, Romania; florinapath@yahoo.com; 5 Thoracic Surgery Department, Dr. Carol Davila Central Military Emergency University Hospital, 020021 Bucharest, Romania; ncd58@yahoo.com; 6 Department 4—Cardio-Thoracic Pathology, Thoracic Surgery II Discipline, Carol Davila University of Medicine and Pharmacy, 020021 Bucharest, Romania

**Keywords:** sternal metastases, manubrium, cancer, biopsy, surgery, chest wall, thyroid cancer, thoracic surgery, breast cancer

## Abstract

We aimed to overview the most recent data on sternal metastases from a multidisciplinary approach (diagnosis strategies, outcome, and histological reports). This narrative review based on a PubMed search (between January 2020 and 22 July 2023) using key words such as “sternal”, “manubrium”, and “metastasis” within the title and/or abstract only included original papers that specifically addressed secondary sternal spreading of cancer in adults, for a total of 48 original articles (14 studies and 34 single case reports). A prior unpublished case in point is also introduced (percutaneous incisional biopsy was used to address a 10 cm sternal tumour upon first admission on an apparently healthy male). The studies (n = 14) may be classified into one of three groups: studies addressing the incidence of bone metastases (including sternum) amid different primary cancers, such as prostate cancer (N = 122 with bone metastases, 83% of them with chest wall metastases), head and neck cancers (N = 3620, 0.8% with bone metastases, and 10.34% of this subgroup with sternum involvement); and glioblastoma (N = 92 with bone metastases, 37% of them with non-vertebral metastases, including the sternum); assessment cohorts, including breast cancer (N = 410; accuracy and sensitivity of PET/CT vs. bone scintigraphy is superior with concern to sternum spreading) and bone metastases of unknown origin (N = 83, including a subgroup with sternum metastases; some features of PET/CT help the differentiation with multiple myeloma); and cohorts with various therapeutic approaches, such as palliative arterial embolization (N = 10), thymic neuroendocrine neoplasia (1/5 detected with sternum metastases), survival rates for sternum metastases vs. non-sternum chest wall involvement (N = 87), oligo-metastatic (sternal) breast cancer (3 studies, N = 16 for all of them), oligo-metastatic head and neck cancer (N = 81), conformal radiotherapy (N = 24,215, including an analysis on sternum spreading), and EBRT followed by MR-HIFU (N = 6). Core data coming from the isolated case reports (N = 34) showed a female to male ratio of 1.6; the females’ ages were between 34 and 80 (mean of 57.28) and the males’ ages varied between 33 and 79 (average of 58.78) years. The originating tumour profile revealed that the most frequent types were mammary (N = 8, all females) and thyroid (N = 9, both women and men), followed by bladder (N = 3), lung (N = 2), and kidney (N = 2). There was also one case for each of the following: adenoid cystic carcinoma of the jaw, malignant melanoma, caecum MiNEN, a brain and an extracranial meningioma, tongue carcinoma, cholangiocarcinoma, osteosarcoma, and hepatocellular carcinoma. To our knowledge, this is the most complex and the largest analysis of prior published data within the time frame of our methods. These data open up new perspectives of this intricate, dynamic, and challenging domain of sternum metastases. Awareness is a mandatory factor since the patients may have a complex multidisciplinary medical and/or surgical background or they are admitted for the first time with this condition; thus, the convolute puzzle will start from this newly detected sternal lump. Abbreviations: N = number of patients; n = number of studies; PET/CT = positron emission tomography/computed tomography; EVRT = external beam radiotherapy; MR-HIFU = magnetic resonance-guided high-intensity focused ultrasound; MiNEN = mixed neuroendocrine-non-neuroendocrine tumour.

## 1. Introduction

Chest wall tumours, including those at the sternum level, account for 5% of all thoracic tumours, and are regarded as challenging conditions that require early recognition and a multitude of surgical approaches for diagnosis through biopsy and resection in order to provide a good cancer-associated outcome [1,2,3,4,5,6]. So far, related statistical evidence is based on small sample studies; a recent expert consensus with regard to their resection and consecutive chest wall reconstruction was released in 2021 [7].

Generally, the prognosis depends on a pluri-factorial panel that takes into consideration the underlying histological report (which is generally found to be very heterogeneous in this particular instance), the tumour-related aggressive profile, and multidisciplinary management of the primary and secondary malignancy, such as surgery, chemotherapy, immunotherapy, radiotherapy, radioiodine treatment, somatostatin analogues, and PRRT (peptide receptor radionuclide therapy) [8,9,10].

When it comes to the sternal involvement originating from non-sternal malignancies (sternum metastases are reported in about 40% of all chest wall metastases), the current level of statistical evidence remains rather low (despite the fact that a few studies, particularly single-centre experiences, have been published) [11,12].

The most common types of published data are either reviews of reported cases concerning sternal metastases originating from a specific histological type of a primary tumour; follow-ups in subjects confirmed with this site of metastases in relationship to different surgical and non-surgical treatments; or the assessments of diagnosis tools, mostly of imaging techniques. Further case-identifying strategies, as well as the surgery candidates’ selection and treatment protocols are required in this difficult matter of metastatic sternum lesions.

We aim to overview the most recent data on sternal metastases from a multidisciplinary perspective, pinpointing various aspects such as the diagnosis strategies, the outcome with respect to different approaches, and the underlying histological reports.

## 2. Methods

This is a narrative review based on a PubMed search (between January 2020 and 22 July 2023) using the key words “sternal metastasis” (strategy 1), “sternum metastases” (strategy 2), and “manubrium metastasis” (strategy 3) within the title and/or abstract. This 3-year sample-based analysis only included original papers that specifically addressed secondary sternal spreading of cancer in adults (originating from any histological type of malignancy), regardless the level of statistical evidence (original studies, as well as case reports). We excluded papers concerning primary malignant tumours of the sternum, studies addressing chest wall tumours that did not provide specific data on the sternal involvement of a malignancy (metastasis), reviews and editorials, experimental studies, and non-English papers.

**Figure 1:**
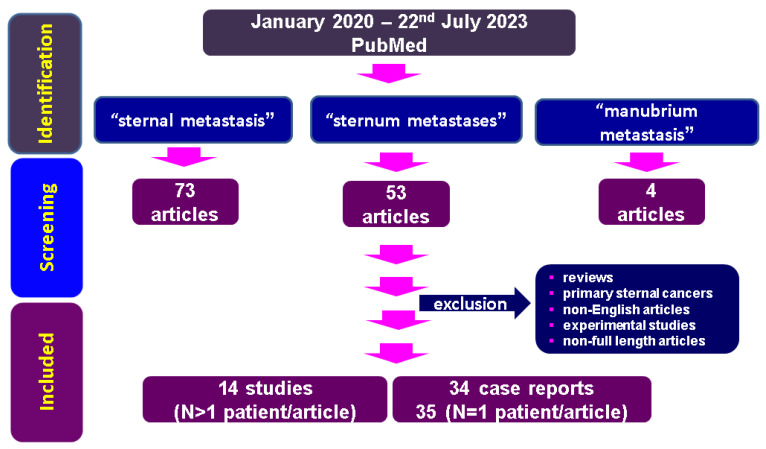
Flowchart of research according to our methods concerning sternal metastases (between January 2020 and 22 July 2023).

According to the three mentioned strategies of research, we identified 73, 53, and 4 articles, respectively. All the papers have been manually checked within the title and abstract for the key words, duplicates were excluded, and, finally, we selected and analysed 48 original articles (14 original studies and 34 single case reports) (Figure 1). A prior unpublished case in point is also introduced as the basis of the discussion in Section 4.

The PubMed search identified a total of 627 articles; after restricting the search to the mentioned timeline according to the three types of key words combinations-based strategies, 130 papers were identified. Following the mentioned inclusion and exclusion criteria, a final number of 48 original papers are included in the final analysis.

## 3. Results: Sternal Metastases—A Multidisciplinary Perspective to Connect the Dots

### 3.1. First Recognition of Sternal Involvement: The Relationship with the Primary Tumour-Associated Histological Profile

Sternal metastases may be related to very frequent malignancies (such as mammary cancer or melanoma) or to unusual primary lesions (from an epidemiologic perspective); however, the rate of sternal involvement is not necessarily correlated with the frequency of the primary cancer among the general population. Generally, the spreading of cancer across the breastbone may be single or multiple (oligo- or multi-metastatic disease) [13,14].

#### 3.1.1. Sternum Metastasis from Primary Malignancies with a High Prevalence in the General Population

Breast cancer is often described in relationship with secondary osseous sites; however, sternal involvement remains rather unusual, especially as a single lesion [15,16,17]. While metastatic breast cancer is a frequent condition, oligo-metastatic disease, particularly to the sternum, is unusual, with the first such report dating back to 1988 [18]. For example, Yao et al. [14] reported the case of a 49-year-old female diagnosed with an aggressive, triple-negative type of cancer that was complicated by pulmonary and sternal metastases that required second-line therapy with immune checkpoint inhibitors (anti-PD-1), in addition to a second regime of chemotherapy (sintilimab, paclitaxel, and carboplatin) following the failure of the initial treatment with paclitaxel and epirubicin [14]. Another interesting report introduced a 73-year-old woman who presented sternal metastases; 9 years had passed since the initial diagnosis, and the patient underwent therapy for the mammary cancer that required radiotherapy and re-starting hormonal treatment. This breastbone complication was followed 2 years later by severe spreading to the epicardium [15]. A similar late, distant metastasis was described in a 74-year-old woman who was found with superficial gastric and colonic metastases 23 years since having surgery for invasive lobular mammary cancer. Additional sternal and vertebral secondary lesions were confirmed on a positron emission tomography/computed tomography (PET/CT) scan [16].

Malignant melanoma, the most frequent dermatologic malignancy (as well as basal cell and squamous cell carcinoma), may spread to any body part, including bone. An exceptional scenario that involved melanoma-related sternum metastasis complicated with dissemination of the tumour thrombus at the level of the internal thoracic vein was reported by Ensle et al. [19] in 2023. This aspect was confirmed by an ^18^F-FDG (18-Fluor-2-desoxi-D-Glucose) PET/CT examination that functions as a very useful imaging tool [19].

A large retrospective study (between 2010 and 2019) on 16,209 subjects with head and neck primary cancers (N = 3620) identified 0.8% of them with bone metastases (N = 29; all the individuals were confirmed with a primary squamous carcinoma and a median period since the first sign of malignancy until bone involvement of 7.8 months was detected), the most frequent site being a vertebra (41%), followed by the pelvis (9%), sternum (10%, N = 3), and femur with a similar prevalence [20]. We identified a second original study on head and neck squamous cell carcinoma. This was a retrospective cohort on 81 patients with oligo-metastatic disease at different sites, including the sternum (admitted between 1998 and 2018); the 5-year survival rate was 40% (N = 32) after multimodal therapy. The results show that the site of metastasis did not influence the survival rate [21].

Alternatively, a bone metastatic score was calculated based on a prediction model in patients with newly diagnosed metastatic prostatic cancer following androgen deprivation therapy as the first -line treatment (N = 122 men; median age of 73; median follow-up duration of 11.5 months). The most frequent skeletal site of disease spreading was the pelvis at 92%, while 83% of them had chest involvement (sternum and ribs) with a hazard ratio (HR) of 2.093; 95% CI: 1.272–3.444, *p* = 0.004 [22].

#### 3.1.2. Breastbone Spreading Originating from Unusual Primary Cancers

A few unusual scenarios have been reported within the time frame of our research, and awareness is necessary among specialists from different medical and surgical domains [23,24,25]. For example, we mention a 70-year-old female who was diagnosed with a primary intra-osseous adenoid cystic carcinoma at the level of the jaw with multiple bone (secondary) spreading, including the sternum. Sasaki et al. reviewed prior published data on this particular histological entity and identified only 51 similar cases [23]. Glioblastoma, a primary brain cancer with a very aggressive profile, rarely complicates with distant spreading. A meta-analysis of Strong et al. included PubMed-published studies of 92 cases with bone metastases (between 1952 and 2021). While the most frequent location was the spine (63%), 37% of these individuals had non-vertebral bone metastasis, including the sternum [24]. Cholangiocarcinoma, a condition that may spread to lymph nodes, the hepatic and pulmonary levels, and even the skeleton, was reported to involve, among other skeletal sites, the sternum of a 60-year-old woman. Singh et al. appreciated this case as the first one of single sternal involvement with respect to this rare malignancy originating from the biliary duct epithelium [25]. Another unusual case was an 80-year-old female with metastatic hepatocellular carcinoma requiring a radical sternum and ribs dissection and further chest wall reconstruction using titanium plates and an acellular dermal matrix [26] (Table 1).

#### 3.1.3. Thyroid Cancer and Sternal Involvement

Thyroid cancer represents the most frequent endocrine malignancy, with a rising incidence during the modern era. Skeletal metastasis of differentiated types, such as papillary and follicular, which represent 80–90% of all thyroid cancers, were reported in 2% to 13% of cases (other reports: 5–10%), follicular being more frequently affected than the papillary form. Skeleton spreading impacts the overall survival rate. The most common sites are the spine, pelvis, sternum, and ribs; usually, bone metastases of these cancers are considered radioiodine resistant, as opposed to other distant metastases, such as pulmonary. Depending on the location of the metastasis, the patients are suitable candidates for surgery and local radiotherapy [27,28,29]. Lately, oral multi-tyrosine kinase inhibitors, such as lenvatinib, have been used in multi-metastatic diseases (including bone) accompanying aggressive tumours [30]. Sternum metastases originating from rarer forms, such as anaplastic or poorly differentiated thyroid malignancies, are only exceptionally reported in association with sternum metastases [31].

One-stage diagnosis of a thyroid (malignant) nodule and associated sternal involvement was described according to the papers we found. For instance, in one case, a 59-year-old female was admitted for synchronous sternal metastases and originating malignancy, namely a papillary thyroid cancer (in combination with an insular carcinoma affecting 40% of the tumour mass). A complex one-stage procedure was performed combining a total thyroidectomy with lymph node resection in association with en bloc resection (a sternal mass of 7 cm) and chest wall rigid reconstruction (titanium bars covered with polymesh dual prosthesis). The thoracic surgery was followed by radioiodine therapy and the patient remained stable for 1 year after surgery while taking levothyroxine suppression medication [28]. Similarly, one case from 2022 involved the diagnosis of a papillary thyroid carcinoma associated with an anterior mediastinal metastatic mass including the sternum (from the notch to the third intercostal space) that required a total thyroid removal and en bloc resection of the sternum metastasis, followed by radioiodine therapy. Stability of the disease was achieved for the following 8 months [32].

Moreover, bone metastases may manifest before the actual recognition/diagnosis of the underlining thyroid condition. We reviewed a case of a 51-year-old female who had a 6-year history of left hip pain impairing her gait. This was caused by a loss of the diaphyseal femur; after 6 years, a check-up showed a large thyroid tumour with tracheal effects (a needle biopsy suggested follicular thyroid neoplasia) in addition to osteolytic lesions at the sternal level and one rib. A total thyroidectomy and neck dissection was performed. A papillary thyroid malignancy was post-operatively confirmed. The patient further underwent ^131^I radioiodine therapy (200 mCi), local radiotherapy, and monthly zoledronate therapy for bone involvement [33].

Of note, small-sized distant metastases from differentiated thyroid malignancies typically respond to standard ^131^I radioiodine therapy, while larger masses are more suitable for surgery; however, an individual decision should be made from a multidisciplinary perspective. This is why, despite a low level of statistical evidence, single lesions of the anterior chest wall might be selectively removed through thoracic surgery (sternal metastasectomy), followed by adjuvant radioiodine therapy [34,35]. Generally, mediastinal metastases from differentiated thyroid carcinomas embrace a more sever prognostic outlook than cervical (neck) recurrence or spreading. For example, the retrospective study of Moritani et al. [36] from 2022 identified 58 subjects diagnosed with papillary thyroid carcinoma who underwent upper mediastinal dissection by sternotomy (the subjects were admitted between 2006 and 2018). A 5-year cancer survival rate of 74.6%, and 10-year survival rate of 58.7% were achieved, which also confirms a more severe prognostic outcome in mediastinal vs. cervical recurrence, while mediastinal metastases larger than 3 cm or lower than the level of the paratracheal nodes were determined to be independent poor prognostic factors [36].

More aggressive histological subtypes are reported as well with regard to sternal metastases; however, the these are only reported in case reports. A sternal metastasectomy was performed on a senior confirmed with a follicular variant of a papillary thyroid cancer with no retrosternal extension, who also had a secondary sternum tumour (of 6 cm) [37]. Similarly, a 33-year-old male was diagnosed with the same histological subtype and, later, he presented with multiple bone metastases (including at the femur diaphysis, ribs, and sternum handle) 2 years after the initial thyroidectomy. In addition to the guided core-needle biopsy of the knee mass, ^18^F-FDG PET/CT assessed the entire bone spreading of the underlying cancer [38]. Hürthle (oncocytic) cell carcinoma, another subtype of differentiated thyroid malignancies with a more severe prognosis due to the fact that it is less radioiodine responsive (or even radioiodine refractory) [39,40], was reported to have distant metastases, including at the mediastinal lymph nodes and pulmonary and sternum levels (lytic lesions). Post-thyroidectomy spreading was identified via FDG PET/CT; the patient received sorafenib for 3 years and then switched to lenvatinib due to hepatic progression of the initial condition [41]. Another unique case was reported of a 79-year-old male who suffered from cutaneous metastasis 16 years after a total thyroidectomy was performed for a benign goitre. The spreading invaded the skin at the level of the upper anterior thoracic wall to the sternal periosteum; thus, a wide excision was performed, in addition to right neck dissection. A right pectoralis major island flap was used for skin reconstruction. Unexpectedly, the histological report showed a papillary thyroid carcinoma with Hürthle cells (which was not consistent with the initial post-thyroidectomy pathological report). The pathogenic traits behind this enigmatic malignant shift are still an open issue [42].

On the matter of the thyroglobulin-producing thyroid carcinoma with unusual sternal involvement, one case of a 59-year-old female was reported to have a negative radioiodine-based whole-body scan after a prior total thyroidectomy and I^131^ radioiodine therapy. Disease relapse in terms of sternal and lung metastases that were iodine-refractory were further detected. Upon exam, they the metastases were determined to be highly positive in somatostatin receptors; thus, PRRT was offered to the patient, and she achieved a thyroglobulin decrease, in addition to an improvement of the sternal pain and respiratory complaints [43]. Currently, PRRT (such as 68Ga/177Lu-DOTATATE) represents an important chapter in the management of iodine-refractory differentiated thyroid malignancies in addition to C cells derivate medullary thyroid cancer (as part of the larger frame, with respect to the neuroendocrine neoplasia); thus, we highlight the importance of determining the somatostatin receptors’ status through Octreoscan and/or immunohistochemistry (if feasible) in radioiodine negative differentiated thyroid carcinomas [44,45].

Recently, genetic analysis in papillary and follicular thyroid cancers (for example, *KRAS*, *BRAF*, *RET*, and *P53* genes) has been considered as a useful tool for identifying an aggressive form, including with a higher risk of distant metastases and/or a radioiodine refractory profile [46]. In 2023, a new report of a recurrent papillary thyroid carcinoma (harboring both *TERT* promoter and *BRAF*^V600E^ mutations) in a 71-year-old woman showed a large recurrent mass involving not only the neck area, but also several local and distant regions, such as the sternal end of the clavicle. [47]. Neoadjuvant treatment, namely, four cycles of anlotinib, a new multi-targeting tyrosine kinase inhibitor [48,49,50], allowed surgical resection by inducing partial tumour shrinkage; however, 6 months following the surgery, a recurrence was noted again [47].

Overall, a heterogeneous interplay between the thyroid and sternum is described. A collaborative team is required for decision-making, while individual management should be sustained by a multidisciplinary check-up.

### 3.2. Advances in Imaging Assessment

As expected, several studies addressed the importance of using PET/CT to assess the bone status, including the sternal metastases [41,51,52,53,54]. A study published in 2023 analysed the accuracy of investigating bone metastases by applying PET/CT vs. traditional bone scintigraphy (N = 410 women with mammary cancer who were followed between 2014 and 2020; 27% of them had associated distant metastases and 72% of these subjects had bone involvement) and identified a higher sensitivity for PET/CT (93.83% vs. 81.84%, *p* = 0.0442) in detecting bone metastases, including the upper and lower limbs, the spine (but not the cranium), as well as the sternum (N = 38 individuals; accuracy of 96% vs. 76% for PET/CT and1 bone scintigraphy, respectively; *p* = 0.0008; sensitivity of 94% vs. 52%, *p* = 0.0001) [51].

A report by Zhao et al. [52] showed that a PSMA (prostate-specific membrane antigen) PET/CT seems more useful for identifying bone metastases (including at the sternum level) than 18 F-FDG PET/CT (in the case of urothelial bladder carcinoma) [52]. A similar conclusion was drawn in a case of sternum metastatic osteosarcoma [55].

Another study on 18 F-FDG PET/CT analysed the bone (osteolytic) metastases in 63 individuals who were not previously diagnosed with a primary cancer; among the studied cohort, 20 patients were finally confirmed with multiple myeloma. Deng et al. [53] analysed the uptake profile for eight distinct skeletal sites (including the sternum). The lesions in multiple myeloma had short cross-section lengths and a more uniform distribution in these 20 subjects vs. the non-myeloma group (N = 43), suggesting the importance of PET/CT detailed features for an adequate interpretation and diagnosis [53].

Single-photon emission computed tomography/computed tomography (SPECT/CT) was found useful in different tumours that are either malignant or hormonally active, such as parathyroid tumours [56,57,58]. Kitajima et al. [56] applied three-dimensional (3D) quantitative bone SPECT/CT to assess the evolution of a patient diagnosed with pulmonary cancer who was treated with pembrolizumab. The initial uptake at the femoral level and left ribs was followed by local radiotherapy for the unilateral femoral head involvement; however, during follow-up, while the uptake was reduced in this site, there was an increased uptake at multiple skeletal sites, including novel sternum spreading [56].

Traditional CT scan and magnetic resonance imaging (MRI) might serve to pinpoint osteolytic lesions [59]. On the other hand, unexpected and undetected (according to a plain X-ray exam) sternal metastases impaired the outcome of a 66-year-old male previously diagnosed and treated for clear cell renal carcinoma; after 5 years of remission, the patient was admitted for aortic valve replacement due to severe stenosis of the bicuspid aortic valve and mitral valve plasty for mitral insufficiency. At the beginning of the cardiac surgery, massive bleeding occurred after the incision was made (at the level of the unsuspected sternal metastasis); in spite of a massive manubrium resection (of 15 cm), in addition to forced ligation of both mammary arteries (as a lifesaving procedure), the outcome was fatal. Approximatively half of the individuals diagnosed with this type of malignancy may present distant spreading, with bone being affected in one-third of them; however, a solitary metastasis, as seen here, is described only in 5% of cases, while in this particular type of kidney cancer, recurrence might occur after a well-established prolonged remission. This is why a check-up with a CT scan, even in asymptomatic subjects, within the remission period is useful before having major surgery [60] (Table 2).

### 3.3. Management of Sternum Metastases

#### 3.3.1. Surgery Notes

En bloc resection of chest wall tumours (with negative margins) represents the gold standard; reconstruction of the chest consecutive to the chest wall defects is mandatory to prevent cardiac, respiratory, and skeletal issues. Various reconstruction techniques with different materials, including 3D technologies for planning, designing, and 3D printing to achieve better surgical guides, have been applied; however, specific guidelines remain an open subject [26,61,62].

Titanium-based bar reconstruction represents a novel alternative, yet, with insufficient statistical evidence [63]. A retrospective analysis was published in 2022 representing the largest single-centre study on this type of malignant tumour with a long-term analysis (a maximum of 108 months). Out of the 87 patients (admitted between 2012 and 2018), 20 individuals had isolated metastases from distant cancers with various origins (such as breast, sarcomas, etc.). Complete resection was performed in 94% of the cases, while a partial sternum resection was performed in 15 subjects (29%). A 5-year survival rate of 57% was confirmed. Overall, the use of titanium bars and sternal plates correlated with a good long-term outcome; however, complications such as a post-surgery infection rate of 18% (mostly, soon after the operation, within the first 12 months, a ratio that seems rather low compared to prior studies) and persistent chest pain (defined as chronic pain for more than 3 months requiring daily medication) affected 24% of the cohort. When compared with the overall survival rate between the subjects with primitive chest wall cancers and secondary malignancies, a long rank *p*-value was not statistically significant (*p* = 0.574) [64].

Generally, neuroendocrine neoplasia represents a difficult dynamic domain; the particular subgroup in the thymic location is a very rare (representing less than 0.4% of all neuroendocrine neoplasia and 5% of all thymic tumours) and aggressive type (that may be typical or atypical carcinoid, large cell neuroendocrine, or small cell carcinomas) [65]. A series (N = 5, male to female ratio of 3 to 2; average age of 53.6 years) published by Huang et al. [66] included a 45-year-old asymptomatic man (N = 1/5) with increased neuron-specific enolase levels in the blood, who was confirmed with stage IV large cell neuroendocrine carcinoma (a mediastinal tumour of 6 cm the largest diameter) with sternum spreading, as well as spreading to other sites, such as the lymph nodes and lungs. The subject underwent tumour resection in addition to the removal of the sternal metastases, upper cava vein, and partial right atrium (with post-operatory remnants at this level) via a median sternotomy and cardio-pulmonary bypass. A post-surgery 4-month survival was associated with rapid progression with distant metastases while being treated with chemotherapy [66]. Limited data have been published so far in order to address the specific field of thymic neuroendocrine tumours; however, a surgical approach is associated with the most significant improvement, including resection of the lymph nodes and distant metastases (if feasible). Additional radiotherapy, chemotherapy, treatment with somatostatin analogues, and PRRT was recommended based on an individual decision [67].

Of note, neuroendocrine neoplasia, particularly the cases displaying carcinoid syndrome originating from gastrointestinal sites, usually correlates with the presence of liver metastases (the liver being involved in 5-hydroxytriptamine metabolism) and, in this case, the patients are candidates for somatostatin analogues, such as octreotide or lanreotide. In spite of this, surgery remains the first choice, if feasible (even debulking procedures might help). In one case of a 59-year-old female with carcinoid syndrome related to a caecum primary tumour, the patient was treated for 6 months with chemotherapy and interferon, followed by Octreoscan-based confirmation of liver and bone metastases (including at the level of the right sternum–clavicle joint); thus, a switch to octreotide LAR was made, allowing for stabilisation of the disease for 2 years when the disease progression required PRRT (177Lu-DOTATATE) [68].

Minimally invasive procedures may also include, for example, percutaneous osteoplasty, as similarly seen in percutaneous vertebroplasty (injection of bone cement). This seems to be a promising alternative to painful sternal metastases, as well, which are no longer responsive to standard therapy, but this aspect is still under debate [69].

#### 3.3.2. Non-Surgical Management of the Sternal Metastases

As an alternative to radiation therapy, or in cases with a poor response to standard management, palliative arterial embolization has been applied. The case series of Papalexis et al. [70] included 10 subjects (male to female ratio of 1, average age of 58.1 years, aged between 37 and 70) diagnosed with sternum metastases from different primary tumours (the patients were admitted between 2007 and 2022). They received palliative arterial Lipiodol embolization (four of them underwent a second procedure). Approximately 90% of them had an occlusion of pathological-associated vessels proven by angiography to correlated with the tumour size reduction, in addition to a clinical improvement, as reflected by the pain scores (according to a median duration of 9.5 months) and a reduction of analgesic medication use [70].

Proton beam therapy was offered to a patient who had prior radiation therapy following a mastectomy for a primary mammary malignancy, according to a report from 2022. The evolution of the malignancy was complicated with a solitary sternum metastasis 6 months after radiotherapy, while the patient was treated with tamoxifen. After experiencing an early complication from this procedure (proton beam therapy-associated dermatitis), a 3-year complete remission was confirmed (in the absence of surgery or chemotherapy, which the subject refused) [71]. Oligo-metastatic mammary cancer with single (isolated) sternum lesions was also the topic of a small study (N = 4) that introduced multimodal treatment (with curative intent) followed by proton pencil beam scanning to the sternum. The disease-free median of the post-diagnosis follow-up was of 28 months [72].

A single-centre experience reported a study on 10 patients with breast cancer who received stereotactic ablative body (3D-conformal) radiotherapy for oligo-metastatic disease at the sternum level. After a median period of time of 32 months, 9/10 subjects had in-field control; among the 7/10 patient with sternal pain, the results after 3 months showed that 3/7 individuals had a decreased pain score and 2/7 subjects had pain remission, suggesting overall a potential analgesic effect. Generally, this type of radiotherapy at the sternum is considered to be more complicated due to the close skin exposure of a larger area [73]. Another large retrospective, multi-centre study on conformal radiotherapy for bone metastases included 24,215 patients (between 2009 and 2016). Sternum spreading was shown, among other sites, to be most likely treated with advanced radiotherapy (OR of 5.2, *p* < 0.001) [74].

Bone pain relief was associated with external beam radiotherapy (EBRT) with good pain control in 60% of cases. In 2021, a first study to address this issue by using magnetic resonance-guided high-intensity focused ultrasound (MR-HIFU) was released; this was a singlearm study on six patients with a median age of 67 (a median lesion of 5.6 cm), who were offered MR-HIFU within 4 days after EBRT. Five of the six subjects registered a good pain response at 7 days, and at 4 weeks, a 60% stabilization rate was registered [75]. Opposingly, a hyper-progression of a sternal metastasis (among other sites) was registered under radiotherapy and immunotherapy, as reported in a case with high-grade urothelial bladder carcinoma [76].

An alternative to surgery for sternal metastases is represented by systemic chemotherapy to target synchronous primary and secondary tumours [77]. For instance, Iijima et al. [78] reported an unusual squamous cell carcinoma on the left side of a 42-year-old woman’s tongue. The histological report was confirmed after lingual ulcer biopsy, while PET/CT confirmed the metastases at the sternum level. She was offered EXTREME regimen (cisplatin and 5-fluorouracil, in association with cetuximab as a loading dose) to be administered six times, then she continued with weekly administration of cetuximab (for 3 years) and achieved sustained remission for 5 years. The authors suggested that the p16 positive status (as revealed by immunohistochemistry exam) was prone to be associated with a better outcome with these drugs [78]. Lately, oral multityrosine kinase inhibitors, such as lenvatinib, have been used in multi-metastatic disease (including bone) accompanying aggressive forms [30] (Table 3).

## 4. Discussion

### 4.1. A Matter of Differential Diagnosis: Sternal Metastases

Differentiating sternum metastases from other entities represent a challenge in some instances. As mentioned, we only included secondary malignancies of the sternum, not primary tumours of breastbone origin, nor their local recurrence [79,80]. It should be noted that chondrosarcoma is regarded as the most frequent primary type (representing 15% of all chest wall tumours), but the issue of primary cancers with sternum origins is very complicated and included unusual tumours, such as multifocal osteosarcomas [81,82,83,84]. Moreover, sternum metastases are distinct from the skin over the sternal area at the metastatic site, although clinical differentiation is not always feasible on the initial admission [85].

Another important differential diagnosis is tuberculosis with bone involvement, which is considered to be a “great mimicker” of a malignancy; however, the sternum is among the rarest sites described under these circumstances, as opposed to, for instance, the spine or hip [86,87]. For example, Engin et al. [87] introduced a case of extra-pulmonary tuberculosis with multiple locations, including the sternum, that initially presented as a suspected malignancy at ^18^F-FDG PET/CT examination. This highlights that awareness is mandatory for differential diagnosis, especially in countries where tuberculosis has an elevated incidence in the general population [87]. Another infectious condition with metastases-like appearance, including at the sternum level, was reported to be alveolar echinococcosis [88].

Interestingly, Yin et al. [89] introduced the case of 42-year-old male confirmed with dermatomyositis after previously experiencing retrosternal pain, rash, and muscle asthenia. After successful corticotherapy, CT showed osteolytic sternum metastases due to a newly detected squamous cell carcinoma of the lungs, as well as tuberculosis, a diagnosis that could not be established at the first presentation for the autoimmune condition [89].

Another distinction should be made in females with mammary cancer that have routine sternum assessments during periodic MRI (or other similar imagery techniques) for disease surveillance [90,91]. Through these assessments, a metastasis at the sternal level may be easily detected, while a sternal incidentaloma underlying, for instance, a hemangioma needs to be differentiated [91].

### 4.2. Insight of a Manubrium Metastasis at First Admission

#### 4.2.1. A Case in Point

**Figure 2:**
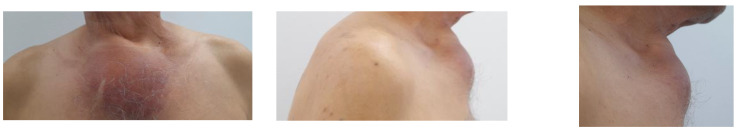
A 61-year-old male admitted for a sternal lump that developed within a few months (different planes).

This was the case of a 61-year-old male who was a heavy smoker and was initially referred to a tertiary centre of endocrinology for a suspected retrosternal goitre. He was clinically stable and had no relevant personal or family medical history. On admission, the clinical exam showed a sternal lump of almost 10 cm with no connection to the thyroid gland. The patient described its progressive increase during the previous few months. He delayed the presentation amid the COVID-19 pandemic (Figure 2).

**Figure 3:**
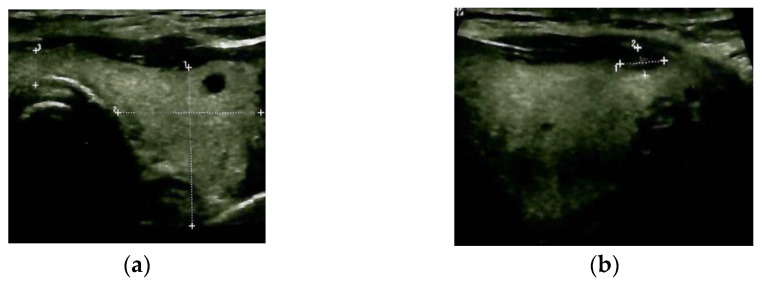
Thyroid ultrasound: multinodular goitre with hypoechoic pattern (**a**): transverse plane; (**b**) (right) sagittal plane.

Blood exams showed mild anaemia in addition to normal thyroid function and negative thyroid antibodies. A thyroid ultrasound revealed a multinodular goitre with a hypoechoic pattern with several nodules of less than 0.6 cm; the largest nodule of 1.1 by 0.6 cm was detected at the upper part of the right thyroid lobe, with no tracheal deviation and no connection to the sternal mass (Figure 3).

**Figure 4:**
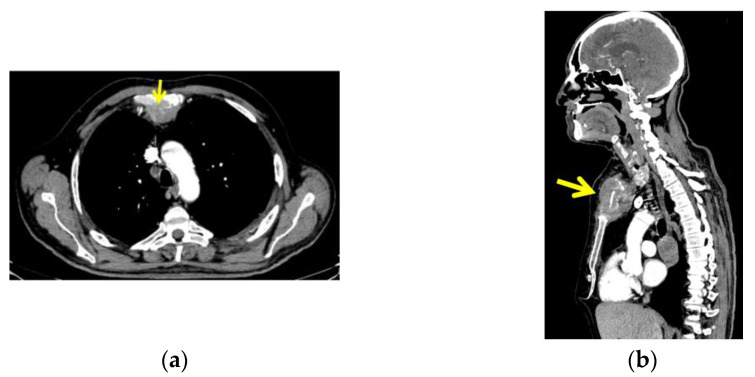
CT with intravenous contrast: sternal tumour of 10 by 11.6 cm; (**a**) transversal plane; (**b**) right: sagittal plane.

CT with intravenous contrast was performed and showed a sternal tumour of 10 by 11.6 cm at the largest diameter, with no retrosternal extension of the goitre (Figure 4). 

The CT scan also revealed two tumours at the level of the dorsal upper pulmonary right lobe, measuring 4 by 3 cm and 3.8 by 3 cm, respectively. Multiple mediastinal lymph nodes and a similar lesion at the level of right lateral cerebellum measuring 3.5 by 2.3 cm, and another at the left temporal lobe measuring 1.9 by 2 cm were also detected (metastases).

**Figure 5:**
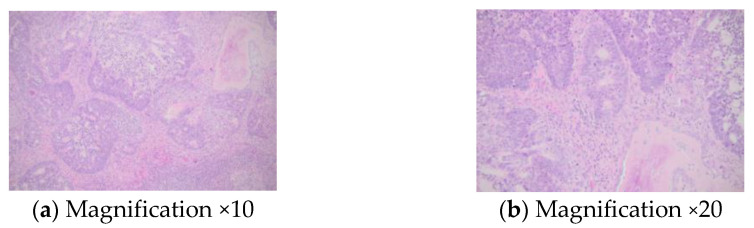
Histological report: sternal metastasis originating from pulmonary adenocarcinoma with a cribriform and glandular pattern; hematoxylin—eosin.

A multidisciplinary decision was made to pursue a biopsy of the sternal tumour in order to provide a histological report; thus, the patient was transferred to the department of thoracic surgery. A percutaneous incisional biopsy of the sternal tumour was performed. After sterile prepping and dressing of the tumour zone, local anesthesia was administered with 10 cc of lidocaine 1%. A small elliptical incision was made, and a tissue sample of 2 by 2 cm was excised. Local hemostasis was achieved with both electrocautery and hemostatic powder. Two 2.0 nylon interrupted stitches were placed. The wound was packed, and the patient was discharged early with no complications. Unfortunately, he died suddenly in his sleep a few days later while experiencing no other clinical complications. The histological report showed lung adenocarcinoma with a cribriform and glandular pattern (Figure 5).

**Figure 6:**
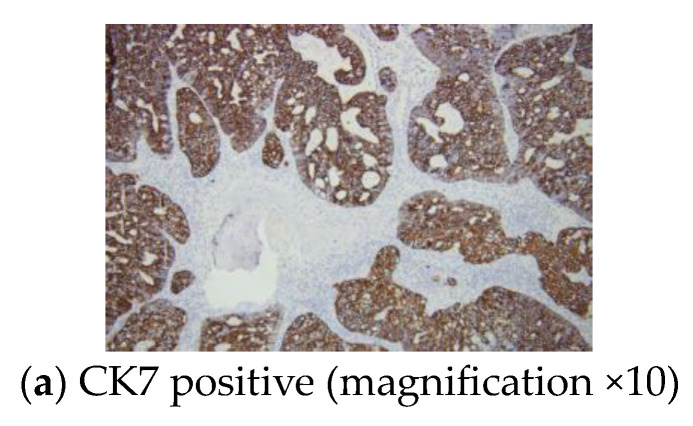

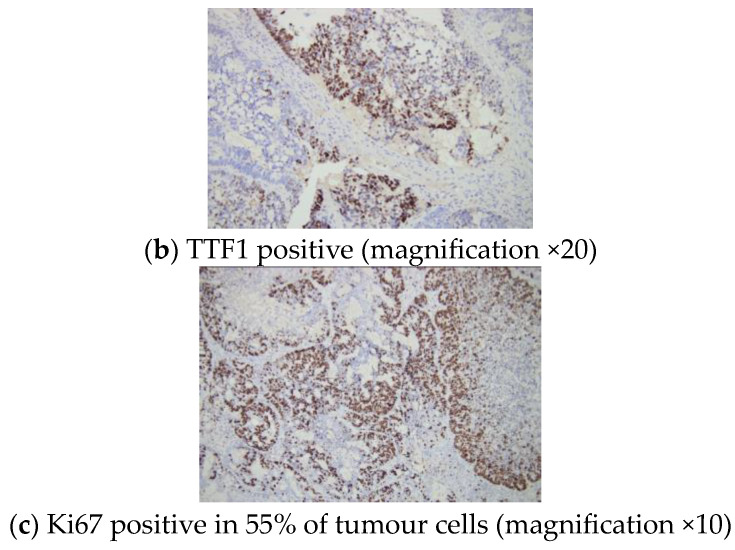
Immunohistochemistry report: sternal metastasis originating from a pulmonary adenocarcinoma with a cribriform and glandular pattern.

Immunohistochemistry analysis revealed a positive CK7 and TTF1 reaction within the tumour cells and a Ki67 proliferation marker of 55% (Figure 6).

#### 4.2.2. Biopsy of Sternal Metastases

According to the data we reviewed and this case, a major player in investigating a newly detected sternal mass is represented by biopsy. Generally, there are several types of biopsies: incisional, excisional (surgical), endoscopic, bone marrow biopsy, fine needle aspiration biopsy, core needle biopsy, punch biopsy, and shave biopsy. The incisional type implies partial tumour removal, while an excisional biopsy removes the entire mass. Fine and core needle biopsies use a smaller or larger needle to collect cells/fluids (fine) or tissue samples (core), with or without imaging guidance. In this case, we performed an incisional biopsy, a surgical procedure in which only a small part of the tumour mass is removed for subsequent pathology examination. It is applied in cases of large, deep-seated tumours, or when a complete surgical excision is not possible because of the medical and surgical risks involved. It can be performed under local or general anesthesia. Percutaneous incisional biopsy of a sternal tumour under local anesthesia is a safe and efficient procedure and can be used in cases when general anesthesia has relative or absolute contraindications. Typically, a biopsy of a sternal mass can be performed by core needle, incisional, or excisional biopsy, depending on the situation. For larger tumours invading adjacent structures, a core needle or an incisional biopsy are preferred. The advantage of the percutaneous core needle biopsy is represented by a reduced risk of hemorrhage and reduced pain and peri-procedural morbidity. Percutaneous incisional biopsy, on the other hand, has the advantage of providing a larger tissue sample for analysis. Excisional biopsy implies the removal of the whole tumour, within oncological limits, and can be performed in tumours up to 5 cm (which was not feasible in this case in point) [23,30,37,62].

In this mentioned case of pulmonary carcinoma with sternal, brain, and lymph node metastases, the patient experienced no local side effects following the biopsy and most probably, the unexpected outcome was related to the brain metastases. As we mentioned, we identified several papers to address biopsy options for sternal masses (within our mentioned methods) [18,23,30,37,62].

Of a similar note, we highlight the case of a 42-year-old male who was admitted for a large mass overlying the manubrium (of 10 by 12 cm). Fine needle aspiration cytology was provided, which was suggestive for a serous cyst; however, after a wide excision was performed, the post-operative histological report confirmed an extra-gonadal seminoma (germ cell tumour). Chemotherapy was administered [92]. This was one of the rarest reports in men diagnosed with seminomas and extra-gonadal involvement [93,94]; however, in the case published by Rathod et al. [92], the spreading mimicked mediastinal involvement, which was not confirmed. Due to the location of the lesion (over the sternum) at the level of soft tissue, fine needle aspiration was a convenient choice, yet it lacked cytological and histological concordance [92].

Another challenging circumstance was described by Hayashi et al. [95]. This was a case of a woman in her 70s who was first admitted for left mammary cancer and axillary lymph node metastases (confirmed via ultrasound-guided biopsy and assessed through ^18^F-FDG-PET/CT). After neoadjuvant chemotherapy, both sites were negative on a subsequent ^18^F-FDG-PET/CT; however, a novel lesion showed a positive sternum accumulation. A sternal (bone) biopsy was performed and found no cancer. The authors suggested that chemotherapy in association with G-CSF (granulocyte colony-stimulating factor) might be responsible for the false positive PET/CT lesions [95]. Direct access via a biopsy avoids unnecessary medication if metastasis is suspected in patients with prior/concomitant non-sternal malignancies [95,96].

Moreover, a CT-guided trans-sternal ^125^I seeds implantation at the level of the mediastinum for primary or secondary tumours at this level represents an additional therapeutic application. A study of 20 patients with 22 different types of lesions (between 2017 and 2019) showed a satisfaction rate concerning the iodine distribution of 90%; a local control rate of 53% was found 1 year after the surgical procedure. In regard to side effects, there was one case of mild pneumothorax and another of hemoptysis [97].

An open biopsy was also used for sternal swelling that developed more than 2 years after the final treatment for a meningioma in a 75-year-old male. The histological report confirmed aetastasis from the initial brain tumour and resection was performed; unfortunately, the disease rapidly progressed to the thoracic cavity and then the abdominal cavity [98]. The same procedure, an open bone (sternum) biopsy was performed on 68-year-old male diagnosed with bladder cancer and osteoblastic lesions with discordant evolution under chemotherapy when compared to the primary site. In cases with multiple skeletal involvements, the decision of performing a biopsy is multidisciplinary, to a certain extent, and it primarily takes into account imaging assessments, such as CT and whole-body bone scintigram, as seen here [99].

Another application of the sternum biopsy was described in a newly admitted adult presenting a 5 cm sternal mass associated with chest pain for 2 months (without a prior history of malignancy). The post-biopsy pathological analysis indicated a renal cell carcinoma as the originating tumour that was complicated by this isolated metastasis. Further targeted therapy was offered to this 76-year-old male [100].

A benign lung and sternum metastasizing leiomyoma was confirmed though biopsy at both levels a decade after a hysterectomy for the originating benign smooth muscle uterine tumour was performed. This most unusual tumour (only a few cases have been previously published), while not being a sarcoma, is slowly growing and hormonally responsive since it displays oestrogen receptors. In this case, therapy with tamoxifen was administrated [101].

A needle biopsy of the sternum was performed for adequate diagnosis following thoracoscopic resection of an exceptional tumour, a primary pulmonary (extracranial) malignant meningioma. The woman presented with persistent tight pain and a further PET/CT was performed and confirmed multiple bone metastases, including the ribs, vertebras, and sternum. Due to the rarity of the initial cancer (only a few cases have been published before this report from 2020), histological confirmation was mandatory; thus, a sternal approach was chosen. These multiple skeletal lesions were then addressed with monthly denosumab and local radiotherapy for femur lesions [102].

As mentioned, the case of an elderly women reported by Pradeep et al. [37] in 2020 involved a diagnosis on first admission of thyroid cancer complicated with manubrium and lung metastases, as highlighted by FDG-PET/CT. For thyroid evaluation, ultrasound-guided fine needle aspiration cytology was performed, while for the sternal mass, the same procedure was applied to confirm the metastases within the thyroid origin. These investigations led to a multidisciplinary decision to perform a total thyroidectomy, unilateral selective neck dissection, and sternal (upper part) metastasectomy (with Dacron mesh, cement, and pectoralis major muscle reconstruction), followed by radioiodine ablative therapy [37] (Table 4).

### 4.3. A 43-Month Sample-Based Study: A Heterogeneous Picture

Overall, we identified 14 studies (of more than 1 subject/article) [18,20,21,22,24,51,53,64,66,70,72,73,74,75] and 34 single case reports [14,15,16,17,19,23,25,26,38,41,42,43,47,52,55,56,59,60,62,68,71,76,77,78,89,98,99,100,101,102] within our methods with respect to sternal metastases (n = 48 papers) (Table 5).

**Figure 7:**
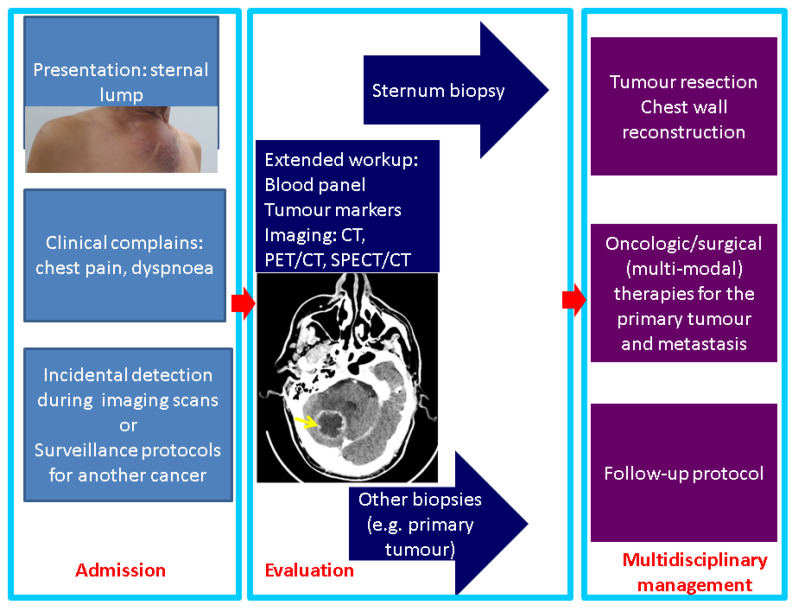
Panel of management in sternal metastases: from presentation to therapy (the photo of the sternal lump represents the case previously described in a 61-year-old male who was admitted for the first time for this breastbone mass, which was finally confirmed as metastatic lung cancer; the image in the second box represents a CT scan with brain metastasis in the same patient).

The case we mentioned brought up the importance of addressing a sternal biopsy into the larger frame of multi-lined, complex management. The detection of sternal metastases might start with the progression of a palpable lump (self-detected by one patient) or with clinical complaints, such as respiratory troubles or chest pain, or it may be evaluated during an imaging assessment for a prior known condition that may or may not be related (incidentaloma) to the sternal mass. Further on, once identified, a tumour of the breastbone should undergo a complex workup and, in selected cases, the decision of performing a sternum biopsy might represent the next logical step of the therapeutic approach (Figure 7).

**Figure 8:**
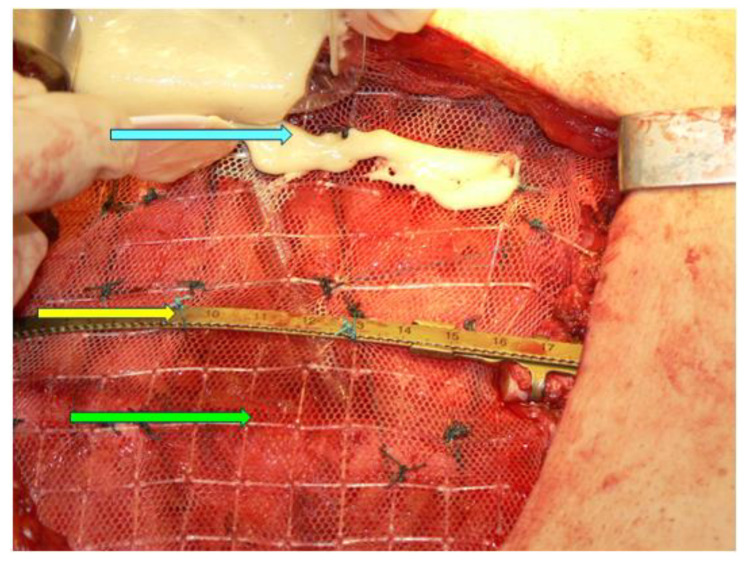
Sternal reconstruction after resection of the medial third of the sternum on an adult patient; Stratos titanium bar (yellow arrow), polypropylene mesh (green arrow), kryptonite bone cement (blue arrow).

Another aspect to pinpoint is the fact that apparently, in this case, a delay of the presentation was related to the recent COVID-19 pandemic. Numerous aspects of medical and surgical practices have been impacted amid this 3-year time frame, and new conditions are being reported or more severe presentations are confirmed due to the deficiency in medical health systems during the dynamic regulations related to the pandemic [103,104]. The impact of prompt, adequate recognition with regard to sternum metastases grossly depends on the underlining primary tumour, the co-morbidities, and the multimodal therapy [105,106]. A multidisciplinary decision is mandatory; yet, in many cases, as mentioned, this decision is personalized rather than being a matter of a specific guideline. Finally, the resection of the mentioned chest wall metastatic tumour and chest wall reconstruction implies another major milestone in decision making and it should be integrated in the overall management in secondary malignant lesions of the breastbone, as seen in primitive sternum cancers [107] (Figure 8).

This sample-related analysis is based on a single database research (PubMed); however, this represents the largest study on published data we could identify. A narrative review allows a more flexible approach since the study design, the studied population, and the assessments/therapies were inhomogeneous. The 14 studies we mentioned may be classified into one of three groups: studies that addressed the incidence of bone metastases (including the sternum) amid different primary cancers, such as prostate cancer (N = 122 with bone metastases, 83% of them with chest wall metastases), head and neck cancers (N = 3620, 0.8% with bone metastases, and 10.34% of this subgroup with sternum involvement), and glioblastoma (N = 92 with bone metastases, 37% of them with non-vertebral metastases, including the sternum); assessments-based cohorts, namely, one on breast cancer (N = 410; accuracy and sensitivity of PET/CT vs. bone scintigraphy is superior with concern to sternum spreading) and another on bone metastases of unknown origin (N = 83, including a subgroup with sternum metastases; some features of PET/CT help with differentiation from multiple myeloma); and the third category is represented by cohorts with various therapeutic approaches, such as palliative arterial embolization (N = 10), thymic neuroendocrine neoplasia-associated management (1/5 subject detected with sternum metastases), survival rates for subjects with sternum metastases vs. non-sternum chest wall involvement (N = 87), oligo-metastatic (sternal) breast cancer (N = 4; in two other studies, N = 10 and N = 2, respectively), oligo-metastatic head and neck cancer (N = 81), applications of conformal radiotherapy (N = 24,215, including an analysis on sternum spreading), and EBRT followed by MR-HIFU (N = 6) [18,20,21,22,24,51,53,64,66,70,72,73,74,75].

**Figure 9:**
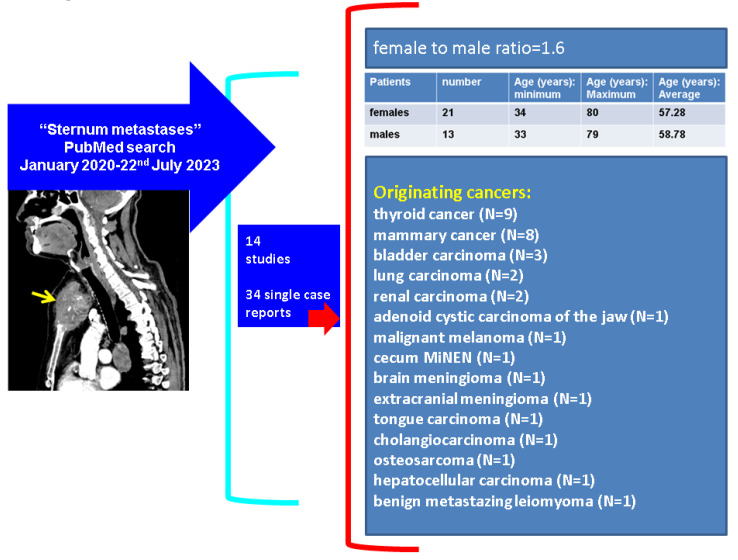
Overview of published case reports (N = 34) according to our methods [14,15,16,17,19,23,25,26,30,32,33,37,38,41,42,43,47,52,55,56,59,60,62,68,71,76,77,78,89,98,99,100,101,102]. Abbreviations: N = number of patients; MiNEN = mixed neuroendocrine–non-neuroendocrine tumour.

The core data coming from the 34 single case reports show the following: female to male ratio of 1:6; the females’ age was between 34 and 80, with a mean value of 57.28 years; and the males’ age varied between 33 and 79 (2 patients were ≤41 years), an average of 58.78 years. The originating tumour profile revealed that the most frequent types were mammary cancer (N = 8, all females) and thyroid cancer (N = 9, both women and men), followed by bladder carcinoma (N = 3), lung carcinoma (N = 2), and malignancy of renal origin (N = 2). One case was identified for each of the following sites: adenoid cystic carcinoma of the jaw, malignant melanoma, caecum MiNEN, brain and an extracranial meningioma, tongue carcinoma, cholangiocarcinoma, osteosarcoma, and hepatocellular carcinoma [14,15,16,17,19,23,25,26,30,32,33,37,38,41,42,43,47,52,55,56,59,60,62,68,71,76,77,78,89,98,99,100,101,102] (Figure 9).

The limitations of the current work are related to a single database search, a 3-year retrospective review, and the analysis according to a narrative review; however, as mentioned, the complexity of the topic and its multidisciplinary approach allowed a wide and insightful standpoint in the matter of breastbone-associated secondary malignancy.

## 5. Conclusions

To our knowledge, this is the most complex and the largest analysis of prior published data within the time frame of our methods. These data open up new perspectives of this intricate, dynamic, and challenging domain of sternum metastases. Awareness is a mandatory factor, since the patients may have a complex multidisciplinary medical and/or surgical background or they are admitted for the first time with this condition; thus, the convolute puzzle will start from this newly detected sternal lump.

## Figures and Tables

**Table 1:** Studies (N > 1 patient per study) focusing on bone metastases incidence (particularly, of the sternum); the table starts with the most recent publication time [20,22,24].

First Author Year of Publication Reference Number	Patients	Results
Zhang 2023 [22]	N = 122 patients with bone metastatic prostate cancer after first-line therapy with androgen deprivation treatment	83% of them had chest wall involvement (sternum + ribs); HR = 2.093; 95% CI: 1.272–3.444, *p* = 0.004.
Gupta 2022 [20]	N = 3620 patients with head and neck cancers	0.8% of them had bone metastases (primary squamous cancers); 10.34% (N = 3) of them had sternum metastases. Average time of survival after bone metastases detection was 5.5 months.
Strong 2022 [24]	N = 92 patients with glioblastoma and bone metastases *	37% of them had non-vertebral metastases (including the sternum, skull, rib cage, and appendicular skeleton).

Abbreviations: N = number of patients; * meta-analysis; HR = hazard ratio; CI = confidence interval.

**Table 2:** Specific studies (N > 1 subject per study) to address the investigations required for sternum metastases diagnosis; the table starts with the most recent year of publication (2023) [51,53].

First Author Year of Publication Reference Number	Patients	Investigation
Cristo Santos 2023 [51]	N = 410 females with breast cancer (mean age of 54 years) sternum metastases (N1 = 38)	PET/CT vs. traditional bone scintigraphy: sensitivity (93% vs. 81%, *p* = 0.0442), similar accuracy (*p* = 0.0775), and specificity (*p* = 0.6) N1 (sternum metastases): accuracy: 96% vs. 76% (*p* = 0.0008) sensitivity 94% vs. 52% (*p* = 0.0001)
Deng 2023 [53]	N = 83 patients with bone metastases from unknown origin (18 F-FDG PET/CT evaluation)	N1 = 20 patients with multiple myeloma N2 = 63 patients with other originating tumours Eight skeletal sites were analysed (including the sternum) N1 vs. N2: shorted cross-section lengths and a more uniform distribution

Abbreviations: N = number of patients; 18 F-FDG PET/CT = 18-Fluor-2-desoxi-D-Glucose positron emission tomography/computed tomography.

**Table 3:** Studies and case series (N > 1 individual per study) aiming to analyse different aspects of management with regard to sternum metastases; the table starts with the most recent year of publication [18,21,64,66,70,72,73,74,75].

First Author Year of Publication Reference Number	Patients	Endpoint
Papalexis 2023 [70]	N = 10 patients with sternal metastases with different origins (mean age of 58.1 years)	Palliative arterial embolization (Lipiodol) causing: ▪ Occlusion for 90% of the pathological vessels; ▪ 50% reduction of the pain score (*p* < 0.005); ▪ Metastatic size reduction during 12 months (*p* < 0.05).
Huang 2022 [66]	N = 5 with thymic neuroendocrine neoplasia C1 = 1/5 with sternum metastases (and other sites)	C1 (large cell neuroendocrine carcinoma): median sternotomy + cardio-pulmonary bypass → tumour resection + removal of sternal metastases, upper cava vein, and partial right atrium → 4-month survival (distant metastases under chemotherapy).
Clermidy 2022 [64]	N = 87 patients with chest wall tumours N1 = 20 patients with sternum metastases (29%)	Similar 5-year survival rate between N1 and non-N1 (*p* = 0.574).
Johnson 2021 [72]	N = 4 patients with oligo- metastatic mammary cancer with single (isolated) sternum metastases	Multimodal treatment (with curative intent) was followed by proton pencil beam scanning to the sternum → median of post-diagnosis follow-up of 28 months was disease-free.
Bartels 2021 [75]	N = 6 patients with bone (sternum) metastases (median age of 67 years)	EBRT followed by MR-HIFU within 4 days → 5/6 subjects registered a good pain response at 7 days, with 60% stabilization at 4 weeks.
Vincent 2021 [21]	N = 81 patients with oligo-metastatic disease (including the sternum) in head and neck squamous cell carcinoma	Multimodal treatment → 5-year survival rate of 40% (N = 32) The site of metastasis does not influence the survival.
Chan 2021 [74]	N = 24,215 patients with bone metastasis and conformal radiotherapy	Sternum metastases were most likely to be treated with advanced radiotherapy (OR of 5.2, p<0.001).
Li 2020 [73]	N = 10 patients with oligo-metastatic disease (at the sternum) in breast cancer	Stereotactic ablative body (3D-conformal) radiotherapy → median follow-up of 32-months: 9/10 patients with in-field control Sternal pain was present in 7/10 patients: 3/7 had improvement of pain + 2/7 had pain remission.
Mohamed 2020 [18]	N = 2 patients with oligo-metastatic disease (at sternum) in breast cancer	Biopsy for each patient (39-year-old and 52-year-old females) → partial and total sternotomy was performed, respectively.

Abbreviations: N = number of patients; C = case; EBRT = external beam radiotherapy; 3D = three-dimensional; MR-HIFU = magnetic resonance-guided high-intensity focused ultrasound.

**Table 4:** Case reports/series addressing sternal or trans-sternal biopsy or fine needle aspiration for diagnosis or therapeutic purposes in non-metastatic lesions of the sternum; the table starts with the most recent publication [92,95,97].

First Author Year of Publication Reference Number	Patient	Lesion at First Presentation	Biopsy	Outcome
Rathod 2023 [92]	42-year-old male	Soft tissue lesion over the manubrium	Fine needle aspiration → cytological report: cystic adenoma (D)	→ wide excision → extra-gonadal metastasis from seminoma (at the level of soft tissue over the sternum)
Hayashi 2022 [95]	Female in her 70s	Positive lesion at ^18^F-FDG-PET/CT after chemotherapy and G-CSF for breast cancer	Sternal (bone) biopsy → no malignancy (D)	False positive lesion at ^18^F-FDG-PET/CT (no sternal metastases)
Wang 2022 [97]	20 patients	Primary and secondary mediastinal tumours	CT-guided trans-sternal ^125^I seeds’ implantation into mediastinum (T)	Satisfaction rate concerning the iodine distribution was identified at 90%; Average time of follow-up was of 12 months; Local control rate of 53% after 1 year; Side effects: 1/20 with mild pneumothorax; 1/20 with hemoptysis.

Abbreviations: D = diagnosis procedure; G-CSF = granulocyte colony-stimulating factor; ^18^F-FDG-PET/CT = 18-Fluor-2-desoxi-D-Glucose positron emission tomography/computed tomography; T = therapeutic procedure.

**Table 5:** Single case reports of sternal metastases according to our methods; the articles are cited starting with the most recent time of publication [14,15,16,17,19,23,25,26,30,32,33,37,38,41,42,43,47,52,55,56,59,60,62,68,71,76,77,78,89,98,99,100,101,102].

First Author Year of Publication Reference Number	Patient	Pathological (Histological) Report of Originating Malignancy	Outcome
Sasaki 2023 [23]	70-year-old female	Primary intra-osseous adenoid cystic carcinoma of the jaw	Biopsy-based histological report; Multiple bone metastases (scapula, sternum,); Chemotherapy → supportive care for 3 years.
Ensle 2023 [19]	76-year-old female	Sternal metastases from malignant melanoma	Spreading of the tumour thrombus at the level of internal thoracic vein (18 F-FDG PET/CT exam).
Yao 2023 [14]	49-year-old female	Triple-negative breast cancer (lung and sternum metastases)	Failure of paclitaxel and epirubicin → 3 cycles of sintilimab + paclitaxel + carboplatin → the patient refused further therapy, thus radiotherapy for lung lesions (+sintilimab) was applied → she refused further chemotherapy and continued sintilimab.
Gao 2023 [47]	71-year-old female	Recurrent papillary thyroid carcinoma *(TERT* promoter and *BRAF*^V600E^ mutations)	Anlotinib (4 cycles) → surgery for extensive recurrence → relapse after 6 months.
Loharkar 2022 [41]	56-year-old female	Hürthle cell (radioiodine resistant) carcinoma thyroid carcinoma	Presentation with distant metastasis (including sternal) → thyroidectomy + neck dissection → 3 years sorafenib → liver metastasis → switch to lenvatinib.
Paspala 2022 [32]	60-year-old male	Papillary thyroid cancer with single large sternum metastasis	Synchronous total thyroidectomy with en bloc sternal metastasectomy → radioiodine therapy (no recurrence for 8-month follow-up).
Polymeris 2022 [68]	59-year-old male	Poorly differentiated MiNEN of the caecum	Octreoscan identification of metastasis at liver, shoulder, sternum-clavicle joint; Therapy: 5-fluorouracil + interferon (6 months) → octrotide LAR 30 mg/month (2 years) → PRRT.
Zhao 2022 [52]	59-year-old male	Urothelial bladder carcinoma	PSMA PET/CT seems more useful for identifying bone metastases (including at sternum) than 18 F-FDG PET/CT.
Yin 2022 [89]	42-year-old male	Squamous cell lung carcinoma	Initial presentation for dermatomyositis; Simultaneous diagnosis of tuberculosis.
Ataei-Nakhaei 2022 [43]	59-year-old female	Thyroglobulin-producing follicular thyroid	Total thyroidectomy → radioiodine therapy → negative disease → disease relapse with sternum and pulmonary metastases (iodine refractory, positive for somatostatin receptors) → PRRT (X2, Lu177 DOTA-TATE).
Ishikawa 2022 [71]	40-year-old female	Breast cancer (oestrogen-receptor positive and progesterone-receptor positive, invasive ductal carcinoma)	Partial mastectomy → radiation therapy → tamoxifen → (after 6 months) single sternum metastasis → proton beam therapy → no recurrence (3-year follow-up).
Rebegea 2021 [15]	73-year-old female	Oligo-metastatic breast cancer with sternal metastasis	Sternal metastasis (9 years since initial diagnosis) → radiotherapy → epicardial metastases (2 years later).
Matsumura 2021 [98]	75-year-old male	Brain meningioma (treated with gamma knife and surgery over a period of 29 years)	Sternal metastases (via open biopsy) → resection of the sternum, ribs, pleura, and pericardium → post-operatory progression into thoracic cavity and pericardium → radiotherapy → progression into abdominal cavity.
Iijima 2021 [78]	42-year-old female	Squamous cell carcinoma of the tongue’s left side (admission with local ulcers)	Biopsy of the lingual ulcer→ PET/CT showed sternal metastasis → EXTREME (cisplatin, 5-fluorouracil, in association with cetuximab as loading dose) for 6 cycles → weekly cetuximab (3 years) → 5-year remission.
Singh 2021 [25]	60-year-old female	Cholangiocarcinoma with bone metastases (including sternum)	Brush biopsy via endoscopic ultrasound was non-diagnostic → biopsy of the iliac crest bone → histological confirmation → palliative cisplatin + gemcitabine.
Dergel 2021 [60]	66-year-old male	Clear cell renal carcinoma	After 5 years of remission, while cardiac surgery was started → bleeding after incision to prior undetected sternal metastasis → manubrium resection → fatal outcome.
Kitajima 2021 [56]	64-year-old male	Pulmonary cancer (treated with pembrolizumab)	SPECT/CT showed uptake at femoral neck and ribs → chemotherapy + local femoral radiotherapy → quantitative bone SPECT/CT showed ↘ femoral uptake + ↗ uptake of other sites (including new sites, such as sternum).
Chen 2021 [59]	42-year-old female	Invasive pleomorphic lobular carcinoma (first admission with bone metastases, including sternal and vertebral fractures on pathologic bone)	Core needle biopsy at mammary tumour → histological confirmation (+immunohistochemistry with HER-2 overexpression, and negative reaction for oestrogen receptor, progesterone receptor) + CT/MRI: multiple osteolytic lesions (including sternum) → chemotherapy + zoledronic acid for bone metastases.
Klonaris 2020 [42]	79-year-old male	Papillary thyroid carcinoma with Hürthle cells	Skin at the level of upper anterior thoracic wall to the sternal periosteum → wide excision + right neck dissection + right pectoralis major island flap for skin reconstruction.
Yue 2020 [77]	34-year-old female	HER2-positive mammary (invasive ductal) cancer	Radical mastectomy at age of 29 → chemotherapy → tratsuzumab (1 year) → lung and sternal metastases at PET/CT→ lapatinib + chemotherapy → disease progression.
Suwardjo 2020 [33]	51-year-old female	Papillary thyroid cancer	Six-year history of left hip pain → loss of diaphyseal femur due to metastases → large thyroid tumour + tracheal deviation → total thyroidectomy + neck dissection → radioiodine therapy → for bone metastases (including sternum) radiotherapy + zoledronic acid.
Kobayashi 2020 [16]	74-year-old female	Invasive lobular mammary cancer	Superficial gastric and colonic metastases 23 years since having surgery for mammary cancer + bone metastases (including sternal).
Pradeep 2020 [37]	“elderly” woman	Follicular variant of papillary thyroid cancer	Ultrasound-guided fine needle aspiration cytology for thyroid and sternum tumours → total thyroidectomy + unilateral selective neck dissection + sternal metastasectomy → radioiodine therapy.
Can C 2020 [55]	75-year-old male	Osteosarcoma with sternum metastases	68Ga-prostate-specific membrane antigen (PSMA) PET/CT showed better radiotracer activity than ^18^F-FDG PET/CT.
Ogasawara 2020 [99]	68-year-old male	Bladder cancer and osteoblastic lesions (including sternum)	Open bone (sternum) biopsy to confirm the metastasis.
Koukouraki 2020 [76]	68-year-old male	Bladder carcinoma	Hyper-progression of a sternal metastasis (among other sites) under radiotherapy and immunotherapy.
Ding 2020 [17]	59-year-old female	Mammary cancer (positive oestrogen receptor status)	Modified radical mastectomy → chemotherapy + radiotherapy → hormonal therapy → recurrence at mammary lymph nodes + sternum → fulvestrant + palliative irradiation.
Yoon 2020 [26]	80-year-old female	Metastatic hepatocellular carcinoma	Radical sternum and ribs dissection → chest wall reconstruction (titanium plates + acellular dermal matrix).
Candanedo-Gonzalez 2020 [38]	33-year-old male	ollicular variant of papillary thyroid cancer	Bone metastases 2 years after thyroidectomy (including sternum handle).
Fernandez 2020 [62]	44-year-old female	Invasive ductal mammary cancer	Manubrio-sternal resection: 3D CT planning for reconstruction + 3D printing of guides for surgical resection → disease-free for the next 12 months of follow-up.
Irzi 2020 [100]	76-year-old male	Renal cell carcinoma with an isolated sternal metastasis on first presentation	Sternum biopsy → histological report: renal cell carcinoma → targeted therapy.
Minami 2020 [102]	67-year-old female	Primary pulmonary (extracranial) meningioma	PET/CT: multiple bone metastasis after thoracoscopic resection of the primary cancer Needle biopsy for sternum metastasis → confirmation of the initial histological report → denosumab (+radiotherapy for femoral metastasis).
Padhi 2020 [101]	47-year-old female	Benign metastasizing leiomyoma	Biopsies of lung and sternum metastases → confirmation of benign leiomyoma → tamoxifen.
Garcia-Rodriguez 2020 [30]	41-year-old male	Follicular thyroid cancer	Hemi-thyroidectomy → 3 years later: PET/CT showed bone metastases (including sternum) → bone (rib) biopsy) → lenvatinib → total thyroidectomy + radioiodine therapy → radioiodine refractory → continue with lenvatinib.

Abbreviations: ↘ = decrease; ↗ = increase; CT = computed tomography; ^18^F-FDG = 18-fluor-2-desoxi-D-glucose; MiNEN = mixed neuroendocrine–non-neuroendocrine tumour; PET/CT = positron emission tomography/computed tomography; PRRT = peptide receptor radionuclide therapy; PSMA = prostate-specific membrane antigen; SPECT/CT = single-photon emission computed tomography/computed tomography.

## Data Availability

Not applicable.

## Abbreviations

CT	Computed tomography
CI	Confidence interval
3D	three-dimensional
EBRT	external beam radiotherapy
^18^F-FDG	18-Fluor-2-desoxi-D-Glucose
HR	Hazard ratio
G-CSF	Granulocyte colony-stimulating factor
MRI	Magnetic resonance imaging
MR-HIFU	Magnetic resonance-guided high-intensity focused ultrasound
PET/CT	Positron emission tomography/computed tomography
PRRT	Peptide receptor radionuclide therapy
PSMA	Prostate-specific membrane antigen
SPECT/CT	Single-photon emission computed tomography/computed tomography
